# Homotopic region connectivity during concussion recovery: A longitudinal fMRI study

**DOI:** 10.1371/journal.pone.0221892

**Published:** 2019-10-02

**Authors:** Catherine D. Chong, Lujia Wang, Kun Wang, Stephen Traub, Jing Li

**Affiliations:** 1 Mayo Clinic Arizona, Phoenix, AZ, United States of America; 2 School of Computing, Informatics and Decision Systems Engineering, Arizona State University, Tempe, AZ, United States of America; University at Buffalo, UNITED STATES

## Abstract

**Objectives:**

To (i) investigate alterations in homotopic functional connectivity (hfc) in concussed patients relative to healthy controls (HC) and to (ii) interrogate whether hfc in concussed patients normalized during the recovery process. The relationship between symptom recovery and change in hfc was assessed using post-hoc analyses.

**Methods:**

This study included 15 concussed patients (mean age = 39.1, SD = 10.1; sex: 13 females, 2 males) and 15 HC (mean age = 39.1, SD = 11.7; sex: 13 females, 2 males). Hfc patterns were interrogated using resting-state magnetic resonance imaging (rs-MRI) for 29 *a priori* selected pain-processing regions. Concussed patients underwent imaging at two time-points; at 1-month post-concussion (mean time following concussion: 28 days, SD = 9.5) and again at 5-months post-concussion (mean time following concussion: 121 days, SD = 13). At both time-points, symptoms associated with concussion were assessed using the Sports Concussion Assessment Tool (SCAT-3).

**Results:**

Concussed patients had significantly weaker hfc in the following six regions 1-month post-concussion compared to HC: middle cingulate, posterior insula, middle occipital, spinal trigeminal nucleus, precentral and the pulvinar. There were no regions of significantly stronger hfc in concussed patients relative to HC. Longitudinally, patients showed significant symptom recovery 5-months post-concussion and had significant strengthening of hfc patterns in seven homotopic ROIs: middle cingulate, posterior insula, middle occipital, secondary somatosensory area, spinal trigeminal nucleus, precentral, and the pulvinar. Post-hoc analyses indicated a significant negative correlation between somatosensory functional connectivity strengthening and symptom severity.

**Conclusion:**

At 1-month post-concussion, patients had significantly weaker hfc in a number of pain-processing regions relative to HC. However, over a period of 5-months, region-pair connectivity showed significant recovery and normalization. Those patients with more successful symptom recovery at 5-months post-concussion had more functional somatosensory strengthening, suggesting an association between functional strengthening and post-concussion symptom recovery.

## Introduction

The prevalence of concussion in the United States exceeds two million people each year, which is a staggering statistic [1]. Concussions are associated with physical, cognitive and emotional sequela. Although these symptoms often resolve over a period of days to weeks, for a significant proportion of patients these symptoms can continue for months or longer [2]. Despite this prevalence of symptoms following concussion, routine diagnostic imaging using magnetic resonance imaging (MRI) or computed tomography (CT) is not sensitive to detect abnormalities related to the concussion injury or indicative of the severity of symptoms associated with a concussion [3, 4]. Research imaging techniques such as resting-state functional magnetic resonance imaging (rs-fMRI) have shown promising results in detecting functional connectivity changes in patients with concussion in a variety of functional networks [5–8], yet the relationship between brain- and symptom recovery following concussion is not clear.

A number of studies have shown strong functional connectivity between equivalent right and left hemisphere regions (homotopic regions), which is thought to underlie stable anatomical connections necessary for efficient information transfer between homotopic brain regions in healthy cohorts [9] [10]. A disruption of homotopic functional connectivity (hfc) has been reported for neurologic disorders including concussion and chronic pain syndromes [11–15]. For example, Sours et al. [16] reported reduced hfc in the dorsolateral prefrontal cortex in patients with high symptom load at one-month post-concussion relative to healthy controls. However, whether disrupted hfc patterns could normalize during concussion recovery is insufficiently investigated.

This study aimed at interrogating hfc in concussed patients relative to healthy controls (HC) in *a priori* selected brain areas associated with pain processing.

Specifically, we interrogated changes in hfc using serial imaging at one-month and at 5-months post-concussion and assessed whether longitudinal changes in hfc related to symptom recovery patterns using post-hoc analyses.

## Methods

### Participants

This study was approved by the Mayo Clinic Institutional Review Board. All research participants over the age of 18 provided informed written consent, prior to study participation. For participants under the age of 18 (n = 1) written consent by the parents and written assent by the participant was obtained prior to study participation. Data from concussed participants included in this study have not been included in other publications. Enrollment criteria included male and female participants ages 16–55. Individuals with concussion were excluded if they had a history of moderate or severe traumatic brain injury. Healthy controls were excluded if they had a history of neurologic disorders including concussion. Concussed patients were recruited from The Department of Emergency Medicine using an operational database that includes all patients seen in the department. The reference database includes demographic information as well as chief complaint, diagnosis, and a record of any central nervous system imaging performed. Healthy controls were recruited within the community.

### Questionnaires

All subjects were screened for TBI using the validated Ohio State TBI Identification method questionnaire [17]. Symptoms of depression were assessed using the Beck Depression Inventory [18]. Symptoms associated with concussion were assessed using the Sports Concussion Assessment Tool (SCAT-3) [19], a self-report questionnaire assessing sensory, cognitive, and emotional symptoms following concussion.

### Image acquisition

Imaging was conducted on a single Skyra Siemens (Erlangen, Germany) 3 Tesla whole-body magnetic resonance imaging scanner using a 20-channel head/neck coil. All imaging was conducted during a time period of 18 months in 2017–2018. No scanner or imaging sequence updates were performed during this time period. Imaging parameters included the followed series:

3D T1-weighted sagittal MP-RAGE (TE = 3.03ms, TR = 2400ms, flip angle = 8°), 128 slices, slice thickness = 1.25mm, 1x1x1.3mm^3^ voxels, 256mm^2^ field of view (FOV), matrix size = 256x256.

Axial T2-weighted imaging were acquired with the following parameters: TE = 84ms, TR = 6800ms, flip angle = 150°, 38 slices with 1x1x4 mm^3^ voxels, slice thickness = 4mm, 256mm^2^ FOV, matrix size = 256x256.

Blood oxygenation level dependent (BOLD) resting-state sequence with TE = 27 ms, TR = 2500 ms, and 4x4x4 mm^3^ voxels, and 256x256 mm FOV.

All structural T1 and T2 imaging scans were reviewed by a board certified neuroradiologist to rule-out gross anatomical abnormalities.

One concussion patient was excluded from further analysis due to abnormal findings on MRI.

#### Resting-state collection and data pre- and post-processing

Ten minutes of blood oxygenation level dependent (BOLD) resting-state imaging data were collected for each participant. Prior to scanning, all participants were instructed to keep their eyes closed but to remain awake, to relax, and to try to clear their minds. All imaging was preprocessed using SPM 8 (Wellcome Department of Cognitive Neurology, Institute of Neurology, London, UK) and the SPM toolbox DPARSF V3.1 advanced edition [20] and interfaced with MATLAB version 11.0 (MathWorks, Natick, MA, USA). All data were processed on a single Macintosh computer (OS X Lion 10.7. software) to avoid postprocessing irregularities that can arise when using more than one workstation.

Standard preprocessing methods included the following: slice-time and motion correction, re-alignment to the first volume, removal of skull and non-brain tissue, spatial smoothing to 4 mm full width and at half maximum (FWHM, Gaussian filter) [21]. To enable signal averaging across all participants, the resting-state images of each participant were first aligned to their own T1-weighted scan and then transformed to the standardized Montreal Neurological Institute (MNI) 305 template. Data were bandpass filtered to between 0.01 to 0.1 Hz to capture low-frequency components of the signal [22]. Signals of no interest (white matter signal, cerebrospinal fluid signal, and global mean signal) were regressed from the data to eliminate noise artifacts due to motion, cardiac, and respiratory rhythms [23]. Variance due to head motion was regressed using a framewise displacement model (FD: threshold for bad time points = 0.5; scrubbing time points before bad time points = 1; scrubbing time points after bad time points = 2) [24]. In addition, prior to including participants in the final analysis, all scans were checked for motion using the DPARSF head motion output file, allowing for data from participants that i) exceeded maximal translation over 2mm or ii) exceeded rotation over 2 degrees in any direction. to be excluded [20].

**Fig 1:**
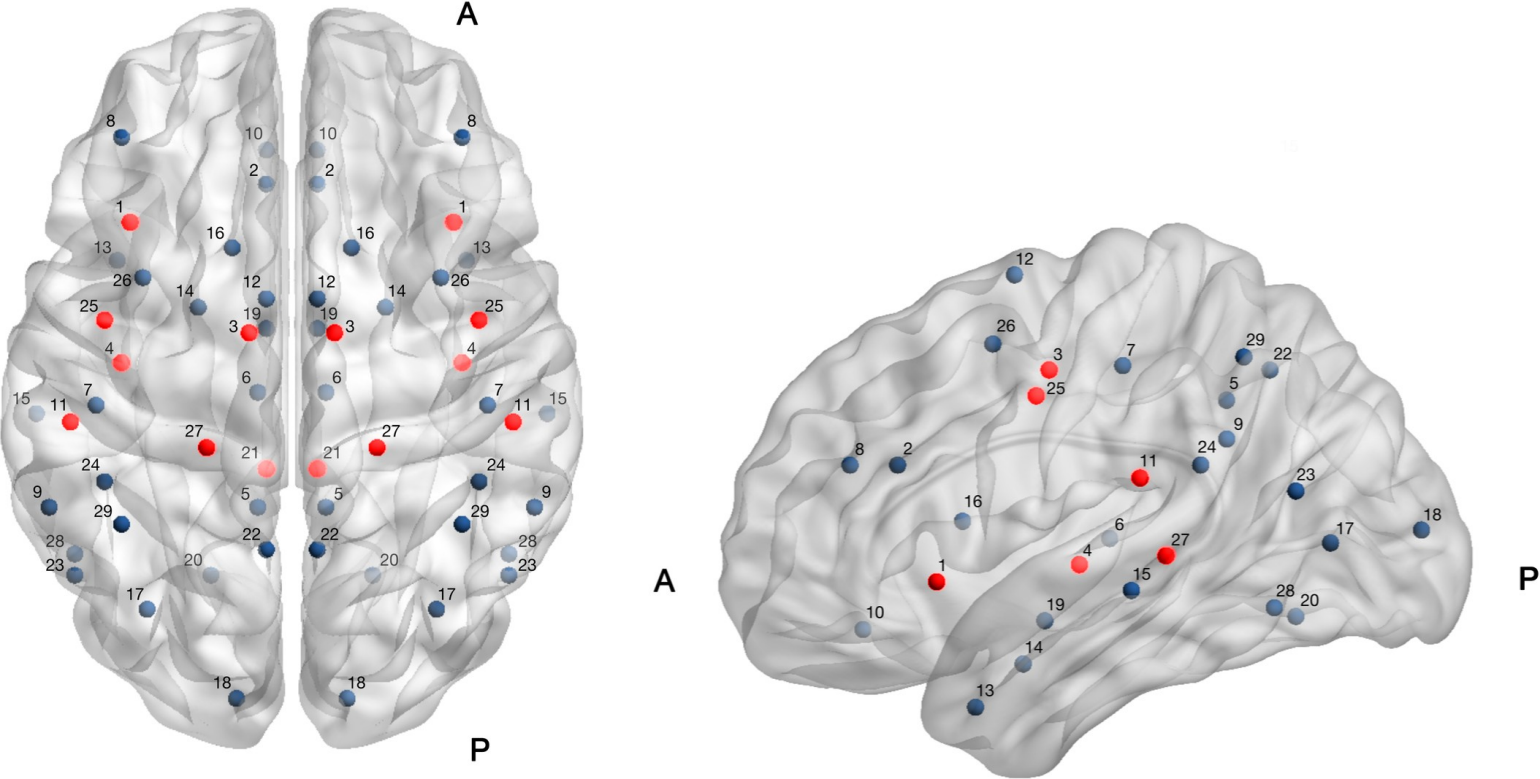
Axial (left) and sagittal (right) brain maps indicateng the approximate location of 29 homotopic (left and right hemisphere) regions of interest (ROIs) on a template brain. A = anterior; P = posterior; ROIs in red indicate homotopic region-pairs with significantly strengthened functional connectivity using serial measurements of functional connectivity at two time-points; 1-month post-concussion to 5-months post-concussion. Those region-pairs that showed significant strengthening (following FDR correction for multiple comparisons per ROI) included the following: 1 = anterior insula; 3 = middle cingulate, 4 = posterior cingulate, 11 = secondary somatosensory, 21 = spinal trigeminal nucleus, 25 = precentral, 27 = pulvinar. ROIs are plotted using BrainNet Viewer (www.nitrc.org).

Functional connectivity (FC) was explored for twenty-nine *a priori* selected homotopic regions-of-interest (ROIs) which were selected based on published literature suggesting that the brain regions a) have atypical functional connectivity or functional activation in prior pain-related studies (including migraine) or b) are typically implicated in pain processing [25–28]. Region names and *x*, *y*, and *z* coordinates are shown in *Table 1* and were determined using the Montreal Neurological Institute (MNI) atlas. ROIs were plotted on a brain template using BrainNet Viewer (www.nitrc.org), *see* Fig 1.

**Table 1:** Twenty-nine left and right-hemisphere regions of interest (ROIs) and corresponding brain coordinates. DLPFC = dorso-lateral prefrontal cortex; VMPFC = ventromedial prefrontal cortex; Inf Lat = inferior lateral; Sup = superior; TPJ = temporo-parietal junction. All x,y,z coordinates are labeled in Montreal Neurological Institute (MNI) space and were explored using an 8-mm sphere. (+/-) indicate right (+) and left (-) hemisphere ROIs. Region names in bolded prints show strengthened functional connectivity in patients over time (1-month to 5-months post-concussion).

ROI	Region Name	*X*	*Y*	*Z*
**1**	**Anterior Insula**	(+/-) 38	19	-3
**2**	Anterior Cingulate	(+/-) 6	28	24
**3**	**Middle Cingulate**	(+/-) 10	-7	46
**4**	**Posterior Insula**	(+/-) 40	-14	1
**5**	Posterior Cingulate	(+/-) 8	-48	39
**6**	Thalamus	(+/-) 8	-21	7
**7**	**Primary Somatosensory**	(+/-) 46	-24	47
**8**	DLPFC (Sup Frontal)	(+/-) 40	39	24
**9**	Inf Lat Parietal	(+/-) 57	-48	30
**10**	VMPFC (Lat Orbitofrontal)	(+/-) 6	36	-14
**11**	Secondary Somatosensory	(+/-) 52	-28	21
**12**	Somatomotor	(+/-) 6	1	68
**13**	Temporal Pole	(+/-) 41	10	-32
**14**	Amygdala	(+/-) 22	-1	-22
**15**	Middle Temporal	(+/-) 60	-26	-5
**16**	Caudate	(+/-) 14	13	11
**17**	Middle Occipital	(+/-) 34	-72	6
**18**	Cuneus	(+/-) 13	-93	9
**19**	Hypothalamus	(+/-) 6	-6	-12
**20**	Lingual Gyrus	(+/-) 19	-64	-11
**21**	**Spinal Trigeminal Nucleus**	(+/-) 6	-39	-45
**22**	Precuneus	(+/-) 6	-58	46
**23**	Parieto-Occipital	(+/-) 51	-64	18
**24**	Supramarginal Gyrus (TPJ)	(+/-) 44	-42	24
**25**	**Precentral (Primary Motor)**	(+/-) 44	-4	40
**26**	Middle Frontal	(+/-) 35	6	52
**27**	**Pulvinar**	(+/-) 20	-34	3
**28**	Fusiform Gyrus	(+/-) 51	-59	-9
**29**	Sup Parietal Lobule	(+/-) 40	-52	49

**Fig 2:**
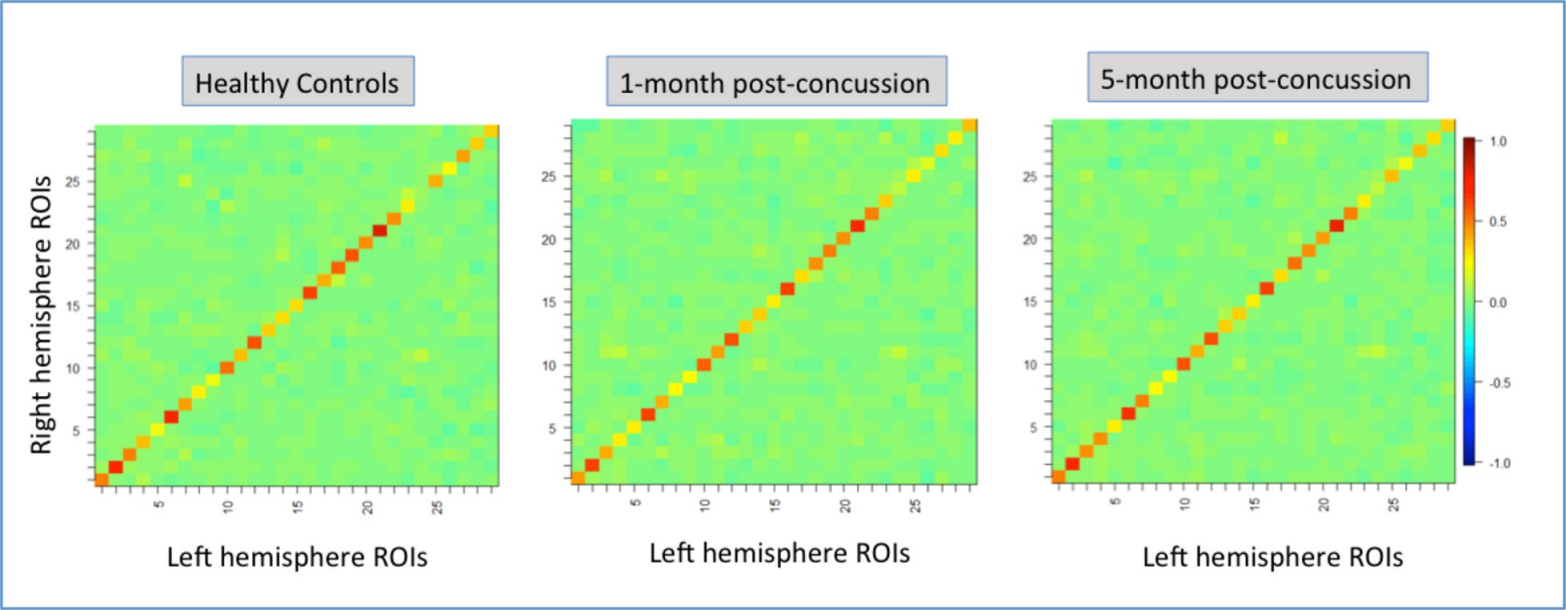
Connectivity matrixes for 29 homotopic (left and right hemisphere) regions of interest (ROIs) for healthy controls, patients 1-month post-concussion and patients 5-month post-concussion. Hot colors (yellow to red colors) indicate positive functional connectivity patterns. Cold colors light blue to dark blue) indicate negative functional connectivity patterns.

Standard post-processing methods included the following: Eight-millimeter spheres were drawn around each of the 29 ROIs, and time courses over each seed region were extracted, and correlation coefficients were converted to a normal distribution via computation of Fisher r-z transformation maps. Connectivity matrixes for 29 homotopic (left and right hemisphere) ROIs are shown in Fig 2.

Functional connectivity was modeled using our previously published Gaussian Graphical Model (GGM) [29]. The GGM took as input the regional time course data of each ROI and estimated an inverse covariance (IC) matrix amongst the ROIs. This method allows estimation of all partial correlations simultaneously and thus avoids the computational burden of computing partial correlations between each of the many pairs of ROIs, consecutively. Within the GGM, a LASSO penalty was integrated for balancing model complexity to a relatively small sample size [30]. The partial correlation (which is inferred from connectivity network directly) for each pair of 29 pre-selected ROIs reflects the connectivity strength between ROIs after factoring out the impact of other ROIs on the correlation.

After the functional network of each subject was built using the aforementioned method, we focused on examining the connectivity strength between the same ROIs on the left and right hemispheres, referred to as “homotopic regions”. For each homotopic region-pair, a FDR-correction and bootstrapping method was used to correct for multiple test comparisons per ROI [31, 32].

#### Differences in hfc between concussed patients at 1-month post-concussion and HC

We calculated the median strength for 15 HC for each of the 29 homotopic ROIs and for 15 concussed patients. A two-sample t-test was performed to assess differences in hfc between concussed patients at 1-month post-concussion and HC.

#### Longitudinal changes in hfc at 1-month compared to 5-months post-concussion

A mixed effect model (MEM) [33] was used to identify longitudinal changes in hfc in concussion patients. Change in connectivity strength over time (1-month compared to 5-months post-concussion) was used as the response variable in the MEM. Another MEM was fit to link connectivity change over time with symptom severity. Demographic differences between HC and concussed patients were interrogated using two-sample t-tests or Fisher Exact tests, as appropriate.

## Results

Eighteen patients with concussion were initially enrolled and scanned at 1-month post-concussion. One concussion patient was excluded from the study due to positive findings on MRI. Two patients had health-related complications unrelated to injury that prevented imaging at 5-months post-concussion and were subsequently excluded from this analysis, thus leaving a total of 15 concussion patients and 15 HC in the final analysis. Concussed patients were well balanced to HC for age and sex and there was no significant difference between groups for age (concussed patients: mean age = 39.1, SD = 10.1; HC: mean age = 39.1, SD = 11.7; *p*-value = 0.84) or sex (concussed patients = 13 females/2 males; HC = 13females/2 males; *p*-value = 1.00). HC without history of concussion were imaged during their normal state of health. Concussed patients underwent imaging at two time-points; 1-month post-concussion (mean time following concussion = 28 days, SD = 9.5) and 5-months post-concussion (mean time following concussion = 121 days, SD = 13.0). *See Table 2*. At both time-points, symptoms associated with concussion were assessed using the Symptom Evaluation component of the Sports Concussion Assessment Tool (SCAT-3), a comprehensive assessment of symptoms commonly used to evaluate sports injuries [34] and a validated self-report questionnaire assessing sensory, cognitive, and emotional pain-related symptoms following concussion [19]. At 1-month post-concussion, patients had a mean symptom severity score of 42.4 (SD = 27.86) and at 5-months follow-up, patients had a mean symptom severity score of 21.6 (SD = 19.9) thus indicating partial symptom recovery over time (p = 0.0017). As a comparison, the HC cohort had a symptom severity score of 2.7 (SD = 6.6).

**Table 2:** Subject characteristics for concussed patients and healthy controls. HC = healthy controls; Concussion *1-month* = average of 30 days post-concussion, Concussion *5-months* = average of 5-months post-concussion; f = female; m = male; BDI = Beck Depression Inventory; SCAT-3 = Sport concussion assessment tool 3; SD = standard deviation; *n/a* = not applicable.

	concussion *(I-month)* *n* = 15	concussion *(5-months)*	HC *n* = 15	*p-value* concussion *(5-months vs 1-month)*	*p*-value concussion *(1-month vs* *HC)*	*p-value* concussion *(5-months vs HC)*
Age, mean (SD)	39.1 (10.1)	*n/a*	39.1(11.7)	*n/a*	.99	*n/a*
Sex (f/m)	12/3	*n/a*	12/3	*n/a*	1.00	*n/a*
BDI (SD)	11.5 (9.1)	10.1(7.8)	1.9 (3.2)	0.25	0.0012	0.0014
SCAT-3 *Symptom severity* (SD)	42.4 (27.86)	21.6 (19.9)	2.7 (6.6)	0.0017	0.000069	0.0028
Number of days post-concussion	28 (9.5)	121 (13.0)	n/a	n/a	n/a	n/a
SCAT-3 *Number of symptoms* (SD)	14.4 (5.7)	9.5 (7.0)	1.7 (3.3)	0.00091	0.00000017	0.00099

Patients had a mean BDI score of 11.5 (SD = 9.1) at 1-month post-concussion and a mean score of 10.1 (SD = 7.8) at 5-months follow-up. Although there were significant differences in the raw scores of the BDI between the concussion cohort and the HC, according to the scoring criteria the mean scores of both cohorts fell into the range of ‘minimal depression’.

Five patients had motor-vehicle related concussions, six patients suffered concussions due to falls, and four patients suffered sports-related concussions. For patients with concussion, four patients had no prior history of concussion, six patients had one prior concussion, two patients had two prior concussions, two patients had 4–5 prior concussions and one patient had 11 prior concussions. For those patients that had a history of multiple concussions, the average time-frame between past and current concussion was 8 years. None of the patients included in the study suffered from persistent post-traumatic symptoms due to a concussion attained in the distant past.

### Patients at 1-month post-concussion versus HC

**Fig 3:**
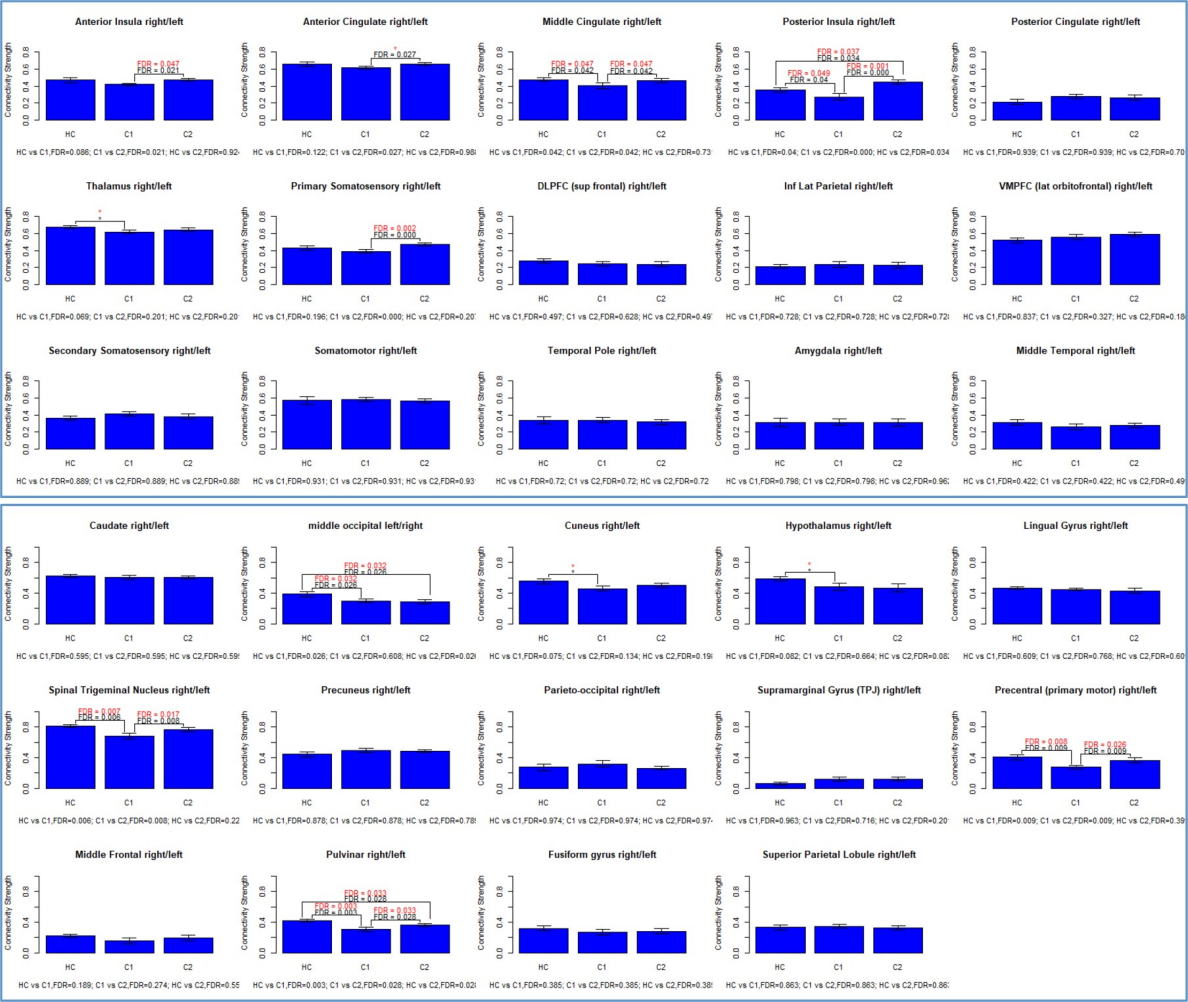
Bar graphs showing the connectivity strength for 29 homotopic regions for healthy controls (HC), concussed patients 1-month post-concussion (time-point CI) and at 5-month post-concussion (time-point CII). P-values in red ink indicate significance after bootstrapping [32].

Relative to HC, concussed patients had significantly weaker functional connectivity in six homotopic ROIs including the: middle cingulate, posterior insula, middle occipital, spinal trigeminal nucleus, precentral (primary motor) and the pulvinar. There were no regions of significantly stronger homotopic connectivity in patients at 1-month post-concussion relative to HC, *see* Fig 3.

### Concussed patients: At 5-months follow-up compared to 1-month post-concussion

Significant longitudinal strengthening of functional connectivity patterns were found in seven homotopic ROIs: middle cingulate, anterior and posterior insula, primary somatosensory area, spinal trigeminal nucleus, precentral (primary motor) and the pulvinar. There were no homotopic regions where functional connectivity was significantly weakened at 5-months compared to 1-month post-concussion.

Functional connectivity between the following homotopic regions showed no significant strengthening or weakening in concussed patients (at either time-point) relative to HC: caudate, lingual gyrus, precuneus, parieto-occipital region, supramarginal gyrus, middle frontal, fusiform, superior parietal, posterior cingulate, dorsolateral prefrontal cortex, inferior lateral parietal, ventromedial prefrontal cortex, secondary somatosensory, somatomotor, temporal pole, amygdala, middle temporal.

### Correlation between longitudinal changes of hfc and symptom severity

Those ROIs that showed significant strengthening in concussed patients over time (1-month to 5-months post-concussion were selected for further post-hoc testing to interrogate relationships between region-pair strengthening and symptom reporting on the SCAT-3. For concussed patients, there was a significant negative correlation between somatosensory functional connectivity strengthening (1-month to 5-months post-concussion) and symptom severity at 5-months post-concussion (r = -0.33; *p* = 0.046).

## Discussion

The hfc of 29 ROIs was explored in HC and in concussed patients 1-month and again 5-months post-concussion. Relative to HC, at one-month post-concussion patients had significantly weaker hfc in six out of 29 pain-related regions, including the following: middle occipital, spinal trigeminal nucleus, precentral, pulvinar, middle cingulate and posterior insula. Serial imaging and concussion symptom evaluation 1-month and 5-months post-concussion showed partial symptom recovery and normalization of hfc in the following seven regions: spinal trigeminal nucleus, precentral, pulvinar, anterior and posterior insula, middle cingulate and primary somatosensory cortex.

Although longitudinal imaging in the concussion cohort indicated a general trend toward hfc normalization in these seven regions, the hfc of the posterior insula in concussion patients was significantly stronger at the 5-month follow-up compared to 1-month post-concussion, and compared to HC. The posterior insula is a region important for sensorimotor integration [35] and has been shown to have altered volume and weaker functional connectivity in concussed patients and in those with chronic pain syndromes including migraine [36–38]. Churchill et al. reported a negative relationship between functional nodal connectivity of the right posterior insula and symptom severity in patients that were imaged acutely following concussion (1–7 days post-injury). Stronger hfc in patients *recovering* from concussion-related symptoms relative to HC is an interesting finding. Whether posterior insula hfc strengthening is indicative of an adaptive or compensatory mechanism to pain [39] will warrant further investigation.

In concussed patients, there was a significant negative relationship between longitudinal primary somatosensory hfc strengthening and symptom severity, thus indicating that patients with better recovery at 5-months post-concussion had more stable somatosensory hfc. The role of the primary somatosensory cortex in pain processing is well-established [40, 41]. For example, Vakhtin et al. reported abnormal somatomotor connectivity in patients with blast-induced concussions (< 3 months post-concussion) relative to HC [42] and Youssef and colleagues reported increased blood flow within the right somatosensory cortex and headache frequency in patients with migraine [43].

### Limitations

Due to sample size limitations, several subanalyses could not be explored as part of this study. (1) As all patients experienced symptom recovery- we were not able to investigate differences in functional connectivity patterns in patients with worsening symptoms versus successful symptom recovery. (2) It was not feasible to investigate the effect of concussion mechanism (motor vehicle accidents vs, sports-related, vs falls) or the number of previous concussions on hfc patterns. Additionally, we did not interrogate whether specific symptoms (such as cognitive, emotional or physical) were related to specific changes in homotopic region connectivity. (3) An unequal male/female (20% male/80% female) ratio in the concussion cohort did not allow for pursuing subanalyses related to sex-related differences between male and female concussion-related brain changes and recovery processes. Future studies, using larger sample sizes will be needed to evaluate recovery from concussion relative to specific concussion populations or symptom sub-domains. In an attempt to focus our analysis to specific ROIs related to pain-processing it is likely that we missed important changes in hfc patterns of regions we left unexplored.

## Conclusion

Relative to HC, concussed patients showed weaker hfc in a number of pain-processing regions at 1-month post-concussion and partial normalization of hfc five-months post-concussion.

Longitudinal imaging in concussed patients indicated greater primary somatosensory hfc in patients with better symptom recovery. These findings may suggest that primary somatosensory hfc could be a potential biomarker for tracking recovery in patients with concussion.
